# Oxygen-Enriched Olive-Oil Dressing in Moderate-Degree Pediatric Burns: Impact on Care and Budget over a 4-Year Period in a Tertiary Children’s Hospital

**DOI:** 10.3390/ebj7010008

**Published:** 2026-02-05

**Authors:** Silvia Borghetti, Ugo Maria Pierucci, Chiara Palladino, Stefania Vimercati, Francesca Selmin, Andrea Marcellusi, Giulia Tosi, Alessia Musitelli, Elena Zoia, Irene Paraboschi, Gloria Pelizzo

**Affiliations:** 1 Pharmacy Unit, ASST Fatebenefratelli-Sacco, 20131 Milan, Italy; borghetti.silvia1@asst-fbf-sacco.it (S.B.); chiara.palladino@asst-fbf-sacco.it (C.P.); stefania.vimercati@asst-fbf-sacco.it (S.V.); giulia.tosi@asst-fbf-sacco.it (G.T.); 2 Department of Pediatric Surgery, “V. Buzzi” Children’s Hospital, 20154 Milan, Italy; alessia.musitelli@unimi.it (A.M.); irene.paraboschi@unimi.it (I.P.); 3 Department of Biomedical and Clinical Science, University of Milano, 20157 Milan, Italy; 4 Department of Pharmaceutical Science (DISFARM), University of Milan, 20122 Milan, Italy; francesca.selmin@unimi.it (F.S.); andrea.marcellusi@unimi.it (A.M.); 5 Anesthesia and Intensive Care Unit, Vittore Buzzi Children’s Hospital, 20154 Milan, Italy; elena.zoia@asst-fbf-sacco.it

**Keywords:** pediatric burns, advanced wound dressing, oxygen-enriched olive-oil dressing, budget impact analysis, hospital costs

## Abstract

Background: Pediatric burns cause considerable morbidity and hospital resource use. Advanced dressings on moderate-degree pediatric burns that accelerate healing may offset acquisition costs by shortening length of stay (LOS). Objective: The aim of this study was to assess the budget impact of introducing an oxygen-enriched olive-oil dressing for pediatric burns (grade I–IIG; total body surface area < 20%) at a tertiary children’s hospital. Methods: A hospital-perspective budget impact analysis was conducted according to ISPOR guidance over a 4-year horizon (2022–2025). The study population included 32 inpatients (<18 years) with non-extensive, moderate-degree burns treated between 2022 and 2023. Two scenarios were modeled: (i) standard of care (SoC) and (ii) SoC plus the oxygen-enriched olive-oil dressing (OEoD), with annual caseload projections to 2025. Costs combined treatment (dressings, drugs, and devices) and hospitalization data provided by the hospital’s Control & Management Unit. The average daily hospitalization cost was €1438.99. Results: Compared with SoC, the OEoD scenario increased per-patient dressing costs (mean €271.4 vs. €121.9) but reduced LOS (mean 7.3 vs. 16.6 days), leading to lower overall hospitalization expenditure. Total annual costs decreased by 7%, 13%, 16%, and 18% across 2022–2025, respectively (for example, 2025: €612,516 vs. €751,445; Δ −€138,929). Cumulative 4-year savings reached €337,399. Deterministic sensitivity analysis confirmed the robustness of these findings, with savings persisting under variable assumptions. Conclusions: Despite higher acquisition costs, oxygen-enriched olive-oil dressings were associated with shorter LOS and meaningful budget savings in pediatric burn care. These results support their integration into multidisciplinary burn management pathways and call for further prospective multicenter validation.

## 1. Introduction

Burn injuries remain a major global health concern, affecting about 11 million people each year and resulting in more than 180,000 deaths, mostly in low- and middle-income countries [1]. Although advances in critical care and infection control have improved survival, burns continue to cause significant morbidity and long hospitalizations, particularly among children [2,3].

In pediatric patients, the impact of burns extends beyond the physical injury. Children are at a higher risk of fluid imbalance, hypothermia, and metabolic derangement due to their greater body surface area-to-mass ratio and immature physiological regulation [4,5,6]. Most pediatric burns occur in domestic settings, frequently due to scalds, and are largely preventable [2,4,7,8,9]. Despite lower total body surface area (TBSA) involvement compared to adults, pediatric burns often necessitate specialized inpatient care to manage pain, wound infection risk, and the psychosocial consequences for both patients and families [10].

Successful burn treatment relies on prompt resuscitation, infection prevention, and optimized wound healing [11,12]. The choice of wound dressing plays a central role in this process. Traditional dressings such as gauze primarily provide mechanical protection and hemostasis, whereas advanced dressings maintain a moist microenvironment, promote re-epithelialization, and reduce bacterial colonization [13,14]. Among emerging technologies, oxygen-enriched oleic matrix dressings have demonstrated a faster healing and antimicrobial activity through the controlled release of reactive oxygen species (ROS) that stimulate angiogenesis and tissue regeneration. The clinical rationale for the oxygen-enriched olive-oil dressing lies in its dual mechanism: the oleic matrix maintains a moist and protective environment, while the controlled release of ROS enhances microcirculation, stimulates fibroblast proliferation, and exerts an antimicrobial effect [15,16]. These biological effects have been documented in both experimental and clinical settings. Bisol et al. in 2022 observed faster epithelialization and decreased inflammation in children treated for pilonidal disease using the same oleic matrix technology [17]. Similarly, in a pediatric cohort, Esposito et al. in 2021 [18] reported that oxygen-enriched oil coatings improved healing rates and reduced postoperative complications after hypospadias repair. Together, these studies support the biological plausibility of the faster wound healing.

Clinical studies in both pediatric and adult populations have reported encouraging outcomes with oxygen-enriched olive-oil dressings, showing reduced inflammation, faster wound closure, and improved patient comfort [17,18,19,20]. However, data regarding their economic impact in the pediatric burn setting remain limited. Evaluating the financial sustainability of introducing advanced dressings is essential due to the increasing emphasis on cost-effectiveness and resource allocation in the hospital system.

The present study aims to assess the economic and clinical implications of introducing an oxygen-enriched olive-oil dressing (OEoD; e.g., NovoX^®^) for pediatric burn care in a tertiary children’s hospital.

## 2. Materials and Methods

This study was designed as a retrospective, hospital-based budget impact analysis (BIA) conducted at Vittore Buzzi Children’s Hospital in Milan, a tertiary referral center for pediatric burns. The analysis was performed according to the guidelines proposed by the International Society for Pharmacoeconomics and Outcomes Research (ISPOR). The evaluation was carried out from the hospital’s perspective, focusing on direct and indirect costs associated with the treatment of pediatric patients with burn injuries.

### 2.1. Study Population

The study population consisted of pediatric patients admitted to the Pediatric Surgery Unit for burn injuries in the years 2022 and 2023. Eligible cases were identified through the Diagnosis-Related Group (DRG) classification system, selecting patients categorized as non-extensive burns without major complications or associated trauma. For the purpose of this analysis, moderate-degree burns were defined as superficial and superficial-partial thickness burns (grade 2–2.5), according to institutional clinical classification. The study population included pediatric patients younger than 18 years of age presenting with non-extensive burns, defined as a TBSA involvement below 20%, for whom complete clinical and administrative data were available to support the economic evaluation. Patients with first-degree burns, deep second-degree or full-thickness burns, extensive burn injuries (TBSA ≥ 20%), or requiring admission to the intensive care unit were excluded. Additional exclusion criteria included the presence of major associated trauma and incomplete medical or cost documentation, which could have compromised the reliability of the analysis.

### 2.2. Study Design

In a 4-year time horizon, from 2022 to 2025, 2 alternative scenarios were compared: the standard-of-care (SoC) scenario represented conventional clinical practice prior to the introduction of the oxygen-enriched olive-oil dressing (OEoD), and the innovation scenario simulating the progressive integration of this new dressing into existing wound management pathways.

Within the innovation scenario, the OEoD (e.g., NovoX^®^ Patch, NovoX^®^ Shield and NovoX^®^ Touch Full Size, MOSS S.p.A., Lesa, NO, Italy) was implemented as an adjunct to the existing standard-of-care pathway, without replacing conventional wound management strategies. In our institutional practice, OEoD is primarily used for superficial second-degree burns, especially when involving functionally or cosmetically sensitive areas such as the face and distal extremities and may also be applied to selected superficial burns in other anatomical regions. The dressing is applied following standard wound cleansing and burn-depth assessment, in conjunction with routine care measures, with the objective of promoting faster re-epithelialization, reducing the frequency of dressing changes, and improving overall patient comfort.

The standard of care includes everything used during the patient’s stay for burn wound care; it therefore includes solutions for cleansing and hydrating the wound, such as those containing betaine or eosin, also used during dressing changes and to prevent the growth of the bacterial biofilm. Different types of creams were used depending on the type of wound and the patient need, in order to maintain a moist wound environment and stimulate the repair and regeneration of the epidermis. Among the dressings used were traditional greasy gauze and, depending on the characteristics of the burn, different advanced dressings. These included polyurethane foam dressings for exuding wounds, as they are highly absorbent, and dressings with silver for antimicrobial action. In 4 cases, a silver nitrate pencil was used to remove excess tissue growths, and in 7 cases, a transparent, vapor-permeable skin substitute was used, as to promote wound healing and reduce the risk of infection.

The model assessed the impact of the 2 approaches on the overall hospital budget by incorporating both direct and indirect costs. The analysis followed the principle of average costing, using aggregated cost data per patient and per hospitalization episode, without extrapolating beyond observed clinical practice.

The analysis was conducted strictly from the hospital perspective. Accordingly, societal indirect costs, such as productivity loss of caregivers or post-discharge non-hospital expenses, were not included. In this context, the term “indirect costs” refers exclusively to hospital-related overheads embedded in the average daily hospitalization cost, including staff, ward-related expenses, and utilities, as calculated and provided by the hospital Control and Management Unit.

Thus, clinical, pharmaceutical, and economic data were collected from hospital medical records, pharmacy logs, and official financial reports. Data from 2022 and 2023 were obtained from the hospital’s Control and Management Unit, while for the years 2024 and 2025, an increasing number of patients to be treated was estimated, and the costs were supposed to be an average of those incurred by the hospital in the previous 2 years.

None of the included patients had a documented history of allergies or skin hypersensitivity reactions, and no cases were excluded on this basis.

### 2.3. Analytical Framework

The analytical model estimated the difference in total hospital expenditure between the 2 scenarios, thereby quantifying the budget impact of adopting the new dressing. The primary outcome was the overall variation in healthcare spending from the hospital perspective, encompassing both inpatient care and wound management costs. All cost components considered in the analysis reflect hospital-incurred expenditures, and no societal or patient-borne indirect costs were included. Secondary evaluations included changes in healthcare resource utilization, such as the average length of stay and the frequency of dressing changes. The analysis aimed to provide a pragmatic assessment of financial sustainability rather than a formal cost-effectiveness comparison. To explore model uncertainty, a deterministic sensitivity analysis was planned, testing the effect of varying key input parameters within clinically and economically plausible ranges.

### 2.4. Ethical Considerations

All procedures performed in this study were in accordance with the ethical standards of the institutional and/or national research committees and with the Declaration of Helsinki and its subsequent amendments. According to the institutional regulations of the Department of Pediatric Surgery, Buzzi Children’s Hospital (Milan, Italy), ethics committee approval was not required for this non-interventional retrospective study based exclusively on anonymized and aggregated data. The confidentiality and reservedness of the collected information were ensured in compliance with Regulation (EU) 2016/679 (General Data Protection Regulation, GDPR) and Legislative Decree No. 101/2018. Informed consent was not required, due to the retrospective nature of the study and the use of fully anonymized data. The OEoD used in this study (e.g., NovoX^®^) is a CE-marked medical device lawfully placed on the European market in accordance with Regulation (EU) 2017/745 on medical devices. It is authorized for routine clinical use in Italy according to its intended use and technical documentation, allowing its adoption in other EU hospitals within standard clinical practice.

### 2.5. Statistical Analysis

Statistical analyses were performed using Stata/BE 18 (StataCorp, College Station, TX, USA). Given the retrospective design, the limited sample size, and the non-normal distribution of several variables, the statistical analysis was primarily descriptive in nature. Continuous variables are summarized using both the median and interquartile range (IQR) as well as mean ± standard deviation, while categorical variables are reported as counts and percentages.

For descriptive comparisons between patients managed under the standard of care and those treated with the oxygen-enriched olive-oil dressing, non-parametric tests were applied for continuous variables (Mann–Whitney U test), and Fisher’s exact test was used for categorical variables. *p*-values were calculated exclusively to assess baseline comparability between groups and to facilitate interpretation of potential confounding factors. No adjustments for multiple testing were performed, and the analyses were not intended to support causal inference or formal hypothesis testing. A two-sided *p*-value < 0.05 was considered statistically significant for descriptive purposes.

## 3. Results

A total of 32 pediatric patients admitted for moderate-degree, non-extensive burn injuries during the study period were included in the analysis. Of these, 22 patients were managed according to the SoC, while 10 received the OEoD as part of the updated wound-care strategy. Baseline demographic and clinical characteristics of the study population are summarized in Table 1.

The two groups were comparable in terms of year of admission, age at hospitalization, sex distribution, burn severity, total body surface area involvement, and etiology of injury, with no statistically significant differences observed. Clinically relevant outcome proxies available from the retrospective dataset, including length of hospital stay and treatment intensity expressed as number of dressings performed, are also reported to provide clinical context for the subsequent economic evaluation.

The economic evaluation was performed from the hospital perspective, combining the costs of treatment and hospitalization. Table 2 summarizes the yearly comparison of expenditures under the two scenarios over the 4-year analytic horizon.

According to Table 2, across the 4 years, total hospital expenditure decreased progressively after the adoption of the new dressing, with the annual percentage savings ranging from 7% in the first year to 18% by the final year. Although the mean cost of treatment per patient increased (from approximately €122 to €271), the reduction in hospitalization costs (driven by shorter LOS) produced a net positive budget impact for the hospital. This progressive reduction reflects the gradual integration of the OEoD into routine clinical practice over time, with an initial use in selected eligible patients followed by broader adoption as clinical familiarity increased, without changes in patient selection criteria or care pathways.

### 3.1. Structure of Hospital Expenditure

Table 3 illustrates the relative weight of each cost component within total expenditure. Hospitalization expenses (including staff, utilities, and ward overheads) represented the vast majority of total costs in both scenarios. The adoption of OEoD marginally increased the proportion of expenditure related to wound dressings but led to a global cost reduction through decreased bed-occupancy time.

### 3.2. Sensitivity Analysis

**Figure 1:**
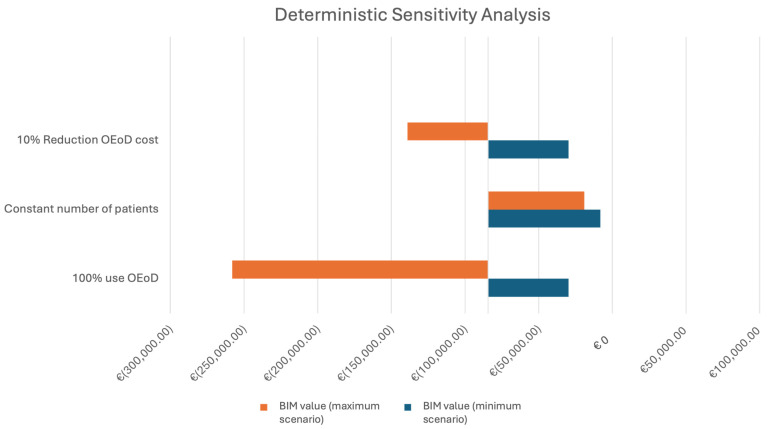
Deterministic sensitivity analysis.

A deterministic sensitivity analysis was conducted to assess the robustness of the model. Some parameters have thus been modified: a 3% reduction in the cost of the OEoD, guessing an inclusion in a regional or independent tender; a constant number of patients treated and a 100% OEoD use. In all those cases, the budget impact analysis is in favor of the new dressing: savings are achieved in all three cases, especially with 100% OEoD use, which can be seen in Figure 1, the tornado diagram.

## 4. Discussion

Burns in children represent a unique therapeutic challenge because of the anatomical, metabolic, and psychological specificities of the pediatric population. As previously reported, pediatric burn injuries are associated with increased morbidity and resource utilization due to a larger surface area-to-body mass ratio, limited thermoregulation, and prolonged healing processes [5,6]. In this context, wound dressings play a central role in influencing recovery, pain, and infection rates.

This study provides one of the first structured budget impact analyses assessing the introduction of an OEoD in pediatric burn care. The analysis demonstrated that, despite higher acquisition costs, the new dressing was associated with a significant reduction in overall hospital expenditure driven primarily by shorter LOS and faster wound healing. Over the 4-year analytic horizon, the cumulative budget saving exceeded €337,000, supporting the economic sustainability of adopting this advanced wound care technology within a pediatric setting.

### 4.1. Economic Interpretation

From an economic perspective, the introduction of advanced dressings often raises concerns regarding the immediate increase in material costs. However, the overall efficiency of burn care depends largely on factors such as LOS, infection control, and need for reoperation, which represent the primary cost drivers in pediatric hospitalization [3]. In our study, the mean LOS decreased from 15.6 to 7.3 days after the introduction of OEoD, corresponding to a reduction of approximately 53%. The shorter LOS reflected faster re-epithelialization and a lower frequency of dressing changes. No local adverse reactions, infections, or intolerance to the new dressing were reported. Patients treated with OEoD required fewer outpatient visits after discharge, and all achieved full wound healing during follow-up. The reduction in hospitalization duration was consistent across age groups and burn etiologies. Hospitalization accounts for nearly 99% of total expenditure, so this improvement translated into a substantial reduction in global costs despite a doubling of treatment-specific expenditure. These results are consistent with previous economic evaluations of advanced wound dressings.

Overall, the adoption of the OEoD in the management of pediatric burns resulted in a shorter average hospital stay, faster wound healing, and lower total hospital expenditure compared with the standard of care. The annual budget impact was consistently favorable across the entire 4-year time horizon, with cumulative savings exceeding €337,399. These findings suggest that the use of advanced oxygen-enriched dressings represents a sustainable and clinically advantageous innovation in pediatric burn care. From an implementation perspective, the observed progressive cost reduction is consistent with the phased adoption of a new wound-care technology, a pattern commonly reported when innovative treatments are gradually incorporated into standard clinical workflows.

The deterministic sensitivity analysis confirmed the robustness of the model: in every tested scenario, the introduction of OEoD remained cost-saving compared with the standard of care.

### 4.2. Limitations

This study has several limitations. First, the analysis was retrospective and single-center, limiting generalizability to other institutions or healthcare systems. Second, the relatively small number of patients reflects the rarity of moderate burns requiring hospitalization in the pediatric population, though this is typical of specialized centers. Third, indirect societal costs (such as parental work absence or post-discharge visits) were not considered; inclusion of these could further strengthen the case for cost savings. Finally, the analysis relied on aggregated cost data rather than individual micro-costing, although sensitivity testing confirmed the internal consistency of results. As with all retrospective, non-randomized studies, selection bias and residual confounding cannot be completely excluded, despite the use of predefined eligibility criteria and consecutive patient inclusion.

### 4.3. Future Perspectives

Future prospective, multicenter studies are warranted to confirm these findings and to explore additional endpoints such as quality of life, pain perception, and patient satisfaction. Combining clinical outcomes with biological markers of wound healing, such as cytokine release, oxidative stress profiles, and angiogenic factors, could further clarify the mechanisms responsible for the accelerated recovery observed with oxygen-enriched dressings [21].

In addition, future studies should include direct comparative clinical and economic evaluations between oxygen-enriched olive-oil dressings and other established advanced wound care materials, such as collagen-based dressings. Particular attention should be paid to differences in acquisition costs, resource utilization, healing outcomes, and patient-centered measures, in order to better define the relative value of these technologies within pediatric burn care pathways.

Beyond these clinical validations, regenerative strategies are rapidly evolving toward biologically integrated wound management. Recent work by Pelizzo et al. in 2018 demonstrated that granulation tissue obtained during burn debridement can serve as an autologous source of mesenchymal stromal cells (GT-MSCs), which display antifibrotic and paracrine properties by modulating TGF-β1 signaling and suppressing fibroblast proliferation [22]. These findings open the possibility of harnessing GT-MSCs as an adjunct or complementary therapy to improve skin regeneration, minimize hypertrophic scarring, and enhance long-term functional and esthetic outcomes in pediatric burns.

Future translational studies could therefore investigate the combination of oxygen-enriched dressings with GT-MSC-based or exosome-mediated therapies, creating a biologically active microenvironment that synergizes the cellular and oxidative components of tissue repair. Integrating such regenerative approaches with robust economic modeling in future clinical trials may help establish formal cost-effectiveness thresholds and guide resource allocation for innovative wound-care technologies in pediatric settings.

## 5. Conclusions

The introduction of the oxygen-enriched olive-oil dressing in pediatric burn management significantly reduced hospitalization duration and overall treatment costs while maintaining excellent healing outcomes and patient safety. Despite higher acquisition costs, the overall budget impact was favorable, confirming the economic sustainability of this innovative wound-care approach. These findings highlight the value of integrating advanced, biologically active dressings into multidisciplinary pediatric burn care to enhance recovery, optimize hospital resource use, and support long-term healthcare efficiency. The study also underscores the importance of collaboration between the hospital pharmacy and clinical teams in the selection and management of wound care technologies. Pharmacists play a strategic role in evaluating cost-effectiveness, standardizing procurement, and ensuring the rational use of resources.

## Figures and Tables

**Table 1:** Baseline demographic and clinical characteristics of the study population.

Variable	Total (N = 32)	SoC (Standard of Care) (N = 22)	OEoD (Oxygen-Enriched Olive-Oil Dressing) (N = 10)	*p*-Value
**Year of admission, n (%)**				0.45
2022	14 (44%)	11 (50%)	3 (30%)	
2023	18 (56%)	11 (50%)	7 (70%)	
**Age at admission, years**				0.58
Median (IQR)	1.7 (1.3–3.0)	1.8 (1.2–2.6)	1.6 (1.4–7.2)	
Mean ± SD	3.3 ± 3.6	2.9 ± 3.3	4.1 ± 4.2	
**Sex, n (%)**				1.00
Male	21 (66%)	14 (64%)	7 (70%)	
Female	11 (34%)	8 (36%)	3 (30%)	
**TBSA (%)**				0.81
Median (IQR)	8.5 (4.5–14.0)	6.5 (5.0–14.0)	10.5 (3.0–14.0)	
Mean ± SD	8.9 ± 5.3	8.6 ± 5.4	9.6 ± 5.3	
**TBSA category, n (%)**				1.00
<5%	8 (25%)	5 (23%)	3 (30%)	
5–15%	22 (69%)	15 (68%)	7 (70%)	
>15%	2 (6%)	2 (9%)	0 (0%)	
**Etiology, n (%)**				0.19
Hot liquids/steam	25 (78%)	17 (77%)	8 (80%)	
Flame	3 (9%)	3 (14%)	0 (0%)	
Heat/vapor	1 (3%)	1 (5%)	0 (0%)	
Contact	1 (3%)	1 (5%)	0 (0%)	
Explosion	2 (6%)	0 (0%)	2 (20%)	
**Total number of dressings**				0.82
Median (IQR)	4.0 (3.0–7.5)	4.0 (3.0–8.0)	4.0 (3.0–5.0)	
Mean ± SD	5.7 ± 4.3	5.9 ± 4.5	5.2 ± 4.1	
**Length of stay (days)**				0.33
Median (IQR)	7.0 (5.0–11.0)	7.5 (5.0–19.0)	6.0 (4.0–10.0)	
Mean ± SD	13.0 ± 21.7	15.6 ± 25.8	7.3 ± 4.2	

**Table 2:** Annual comparison of hospital costs and budget impact between the SoC and OEoD scenarios.

Year	Cost Component	SoC (Standard of Care) (€)	OEoD (Oxygen-Enriched Olive-Oil Dressing) (€)	Budget Impact (€)	Variation (%)
**2022**	Treatment cost	1707	2155	−448	+26%
	Hospitalization cost	402,917	372,698	−30,219	−7%
	**Total**	404,624	374,853	−29,771	**−7%**
**2023**	Treatment cost	2195	3241	−1046	+48%
	Hospitalization cost	518,036	447,526	−70,510	−14%
	**Total**	520,231	450,767	−69,464	**−13%**
**2024**	Treatment cost	2682	4177	−1495	+56%
	Hospitalization cost	633,156	532,426	−100,730	−16%
	**Total**	635,838	536,603	−99,235	**−16%**
**2025**	Treatment cost	3170	5262	−2092	+66%
	Hospitalization cost	748,275	607,254	−141,021	−19%
	**Total**	751,445	612,516	−138,929	**−18%**

Note: The introduction of OEoD increased treatment expenditure but substantially reduced hospitalization costs, leading to an overall cumulative saving of €337,399 over four years. Percentage variations refer to the relative change compared with the standard of care.

**Table 3:** Distribution of hospital expenditure by cost component.

Cost Component	SoC Scenario	OEoD Scenario	Change (%)
Wound-treatment materials (dressings, drugs, devices)	0.4%	0.7%	+0.3%
Hospitalization (ward stay, staff, overheads)	99.6%	99.3%	−0.3%
Total	100%	100%	–

## Data Availability

The datasets presented in this article are not available, because all underlying data are derived from hospital administrative and clinical systems and are therefore strictly sensitive.
